# The synergistic necrohemorrhagic action of *Clostridium perfringens *perfringolysin and alpha toxin in the bovine intestine and against bovine endothelial cells

**DOI:** 10.1186/1297-9716-44-45

**Published:** 2013-06-19

**Authors:** Stefanie Verherstraeten, Evy Goossens, Bonnie Valgaeren, Bart Pardon, Leen Timbermont, Karen Vermeulen, Stijn Schauvliege, Freddy Haesebrouck, Richard Ducatelle, Piet Deprez, Filip Van Immerseel

**Affiliations:** 1 Department of Pathology, Bacteriology and Avian Diseases, Faculty of Veterinary Medicine, Ghent University, Salisburylaan 133, Merelbeke B-9820, Belgium; 2 Department of Internal Medicine and Clinical Biology of Large Animals, Faculty of Veterinary Medicine, Ghent University, Salisburylaan 133, Merelbeke B-9820, Belgium; 3 Department of Surgery and Anesthesia of Domestic Animals, Faculty of Veterinary Medicine, Ghent University, Salisburylaan 133, Merelbeke B9820, Belgium

## Abstract

Bovine necrohemorrhagic enteritis is a major cause of mortality in veal calves. *Clostridium perfringens* is considered as the causative agent, but there has been controversy on the toxins responsible for the disease. Recently, it has been demonstrated that a variety of *C*. *perfringens* type A strains can induce necrohemorrhagic lesions in a calf intestinal loop assay. These results put forward alpha toxin and perfringolysin as potential causative toxins, since both are produced by all *C*. *perfringens* type A strains. The importance of perfringolysin in the pathogenesis of bovine necrohemorrhagic enteritis has not been studied before. Therefore, the objective of the current study was to evaluate the role of perfringolysin in the development of necrohemorrhagic enteritis lesions in calves and its synergism with alpha toxin. A perfringolysin-deficient mutant, an alpha toxin-deficient mutant and a perfringolysin alpha toxin double mutant were less able to induce necrosis in a calf intestinal loop assay as compared to the wild-type strain. Only complementation with both toxins could restore the activity to that of the wild-type. In addition, perfringolysin and alpha toxin had a synergistic cytotoxic effect on bovine endothelial cells. This endothelial cell damage potentially explains why capillary hemorrhages are an initial step in the development of bovine necrohemorrhagic enteritis. Taken together, our results show that perfringolysin acts synergistically with alpha toxin in the development of necrohemorrhagic enteritis in a calf intestinal loop model and we hypothesize that both toxins act by targeting the endothelial cells.

## Introduction

Since the ban on antimicrobial growth promoters in Europe, necrohemorrhagic enteritis emerged as a major cause of mortality in veal calves in Belgium, causing important economic losses [1-3]. Bovine necrohemorrhagic enteritis, also known as enterotoxaemia, is most typically characterized by sudden death and macroscopic post-mortem findings are necrotic and hemorrhagic lesions in the small intestine [4,5]. Microscopically, necrosis of the intestinal mucosa and hemorrhages are observed [1,5,6]. *Clostridium perfringens* is considered as the causative agent [1,7]. This spore-forming, Gram-positive, anaerobic bacterium is often found as a normal inhabitant of the intestine of most animal species and humans [6,8,9]. For reasons that are not yet fully understood, *C*. *perfringens* can, under certain predisposing conditions, proliferate rapidly, concurrently produce toxins and cause disease. Stress, for example, is considered to be such a predisposing factor, and also the feed composition is believed to be of major importance for the development of the disease [7,10,11]. The presence of a causative toxin determines the potential of a *C*. *perfringens* strain to cause lesions in several animal species. Classification of *C*. *perfringens* strains is based on the production of four major toxins, namely alpha, beta, epsilon and iota toxin. In addition to the major toxins, other toxins can be secreted, such as beta2-toxin and perfringolysin [5,12].

There has been controversy on the toxins responsible for bovine necrohemorrhagic enteritis. Some studies proposed epsilon toxin as a possible causative toxin [13-16]. This toxin is produced by type B and D strains and plays a key role in the pathogenesis of sheep and goat enterotoxaemia. Filho et al. could indeed induce clinical signs and lesions, when a type D strain was inoculated intraduodenally in a calf [15]. In an intestinal loop assay comparing alpha and epsilon toxin, only epsilon toxin was able to cause severe oedema and hemorrhages in the lamina propria [9]. These results point to epsilon toxin as causative toxin. More recently, beta2-toxin (CPB2) has been linked to necrohemorrhagic enteritis in calves and cows [2,6,17-19]. Manteca et al. showed that inoculating a beta2-positive type A strain into a bovine ligated intestinal loop caused hemorrhages of the intestinal wall and necrosis [19]. The strain however also produced high levels of alpha toxin, so a synergistic action between both toxins was proposed. Moreover, allelic variants of the CPB2-gene have been identified and strains from cattle mostly carried the atypical CPB2-gene that is not expressed, in contrast to the “consensus” variant [2,6,17]. In addition, a more recent study isolated only beta2-negative type A strains from calves with necrohemorrhagic enteritis and these strains were also able to induce pathological changes in inoculated intestinal loops [10]. We recently developed an experimental intestinal loop model that mimics the typical lesions of calf necrohemorrhagic enteritis both macroscopically and microscopically [20]. In this model, it was demonstrated that type A strains from bovine and non-bovine origin, including both beta2-negative and -positive strains, were able to induce necrohemorrhagic lesions [20]. These results suggest that the causative toxin should be present in all the tested *C*. *perfringens* strains. This puts forward a potential role for alpha toxin and perfringolysin, since both are produced by nearly all *C*. *perfringens* strains [21]. The importance of perfringolysin in the pathogenesis of bovine necrohemorrhagic enteritis is not reported before. In gas gangrene or clostridial myonecrosis, perfringolysin is involved in the pathogenesis in synergy with alpha toxin [22-24]. Additionally, Valgaeren et al. observed that the early pathogenesis of calf necrohemorrhagic enteritis in an experimental loop assay is characterized by congestion of the capillaries resulting in hemorrhages and finally necrosis of the mucosa [20]. This points to the endothelium as a possible target.

Therefore, the objective of the present study was to evaluate the role of perfringolysin in the development of necrohemorrhagic enteritis and its synergism with alpha toxin in calves, by using a calf intestinal loop assay and endothelial cell cytotoxicity assays.

## Materials and methods

### Bacterial strains and culture conditions

The bacterial strains used were a wild-type gas gangrene strain (JIR325) and its isogenic mutant genetically deficient in the production of perfringolysin and alpha toxin (Δ*pfoA* Δ*plc* double mutant JIR4444) as well as the Δ*pfoA* Δ*plc* double mutant complemented with a plasmid that carried either the perfringolysin gene (*pfoA*-complemented Δ*pfoA* Δ*plc* mutant JIR4460) or the alpha toxin gene (*plc*-complemented Δ*pfoA* Δ*plc* mutant JIR4461) or both toxins (*pfoA plc* double-complemented Δ*pfoA* Δ*plc* mutant JIR4462) (Table 1) [23,25]. All strains were kindly provided by J.I. Rood (Department of Microbiology, Monash University, Clayton, Victoria, Australia). The strains were grown at 37 °C in Brain Heart Infusion (BHI) broth (Oxoid, Basingstoke, United Kingdom) with 0.375% glucose in an anaerobic (84% N_2_, 8% CO_2_ and 8% H_2_) cabinet (Ruskinn Technology, Bridgend, UK). When required, chloramphenicol (30 μg/mL) was added to the growth media. The supernatants were filter-sterilized over a 0.2 μM filter before use in the experiments (Merck Millipore, Billerica, Massachusetts). When a logarithmic-phase culture was used, a 1/100 dilution from an overnight culture was made 3 h prior to use and was incubated in an anaerobic cabinet.

**Table 1:** Description of strains and toxin activities in supernatants of an overnight culture

**Strain**^**a**^	**Strain number**	**Relevant characteristics**^**b**^	**Plasmid-encoded toxin gene(s)**^**a**^	**Plc activity(μg mL**^**-1**^**)**^**c**^	**PFO activity (log**_**2**_**(titre))**^**c**^	**Ref**.
Wild-type	JIR325	Strain 13^d^		12.5 ± 2.5	4.8 ± 0.2	[25]
Δ*pfoA*Δ*plc*	JIR4444	JIR325 pfoA::ermB plcΩpJIR1774, suicide plasmid		< 1.0	< 1.0	[23]
*pfoA*-complemented Δ*pfoA*Δ*plc*	JIR4460	JIR4444(pJIR871), Cm^R^	*pfoA*^+^	< 1.0	4.1 ± 0.3	[23]
*plc*-complemented Δ*pfoA*Δ*plc*	JIR4461	JIR4444(pJIR1642), Cm^R^	*plc*^+^	6.8 ± 0.7	< 1.0	[23]
double-complemented Δ*pfoA*Δ*plc*	JIR4462	JIR4444(pJIR1720), Cm^R^	*pfoA*^+^p*lc*^+^	8.0 ± 0.5	3.5 ± 0.1	[23]

^a^*pfoA:* perfringolysin gene, *plc* : alpha toxin gene, ^*b*^*Cm*^r^: chloramphenicol resistant, ^*c*^*Plc* : alpha toxin, PFO: perfringolysin O, ^d^JIR325 is a rifampicin and nalidixic acid resistant derivative of strain 13, a gas gangrene strain.

Filter-sterilized supernatant of an overnight culture of each strain was assayed for perfringolysin activity by the doubling dilution hemolytic assay with horse red blood cells as described previously (Table 1) [26]. The perfringolysin activity was expressed as the reciprocal of the last dilution which showed complete hemolysis. The perfringolysin assay was performed in triplicate. For the detection of alpha toxin in the supernatant, the Bio-X Alpha Toxin Elisa Kit (Bio-X Diagnostics, Jemelle, Belgium) was used according to the instructions of the manufacturer (Table 1). The amount of alpha toxin was calculated as described previously in μg mL^-1^[27]. The alpha toxin ELISA was performed in triplicate.

### Assessing the role of perfringolysin by using an intestinal loop model

The animal studies were undertaken with approval (EC2012_056) of the Ethical Committee of the Faculty of Veterinary Medicine (Ghent University). Three 11-week-old Holstein Friesian calves, originating from a commercial veal herd, were used. Intestinal loop assays were conducted as described by Valgaeren et al. [20]. Briefly, the calves were anesthetized and the small intestine was exteriorized. Intestinal loops were ligated and injected with 20 mL logarithmic cultures followed by an injection of 10 mL 25% commercial milk replacer (Vitaspray, Vitamex®, Drongen, Belgium) suspended in sterile 0.9% NaCl solution. Because the gas gangrene strain JIR325 could induce lesions comparable to the bovine strains, this strain was used in combination with its isogenic mutants (Table 1) [20]. Each strain was injected in quintuplicate and as a control BHI was injected in five loops instead. After injection of the loops, the abdomen was closed and the calves were maintained under anesthesia. At 6-h post-inoculation the animals were euthanized and samples were taken. Samples were fixed in 4% phosphate buffered formaldehyde. They were embedded in paraffin wax, sectioned and stained with hematoxylin-eosin by the conventional method for histological examination. The sections were evaluated for presence of necrotic lesions (Leica DM L2 microscope with Leica DFC320 camera and LAS software).

### Isolation of bovine umbilical vein endothelial cells

Primary bovine umbilical vein endothelial cells (BUVEC) were isolated from umbilical cord veins by an adaptation of the method of Jaffe et al. [28]. Umbilical cords obtained from calves born by caesarean section, were transported in 0.9% HBSS-HEPES buffer (pH 7.4, Gibco, Grand Island, NY, USA) supplemented with 500 U/mL penicillin (Sigma-Aldrich) and 50 μg/mL streptomycin (Sigma-Aldrich). The umbilical vein was cannulated and flushed with pre-warmed (37 °C) HBSS (Gibco, Grand Island, NY, USA). 0.5% collagenase type IV (Sigma-Aldrich) suspended in HBSS was infused into the lumen of the clamped shut umbilical vein and it was incubated for 30 min at 37 °C. After gently massaging the umbilical vein, the cells were flushed from the vein by perfusion with 10 mL HBSS containing 20% fetal calf serum (FCS, Bockneck Labs Inc., Toronto, Canada). After rinsing (250 × g, 5 min), a single cell suspension was obtained by filtration through a 70 μm cell strainer (BD Labware, San Jose, CA, USA). The pelleted (250 × g, 10 min) cells were resuspended in endothelial cell growth medium containing 20% FCS (EGM-2; Lonza, Basel, Switzerland) and seeded in 25 cm^2^ plastic tissue culture flasks. After 24 h incubation at 37 °C in the presence of 5% CO_2_, the cell medium was changed. Bovine umbilical vein endothelial cells were grown to confluence. The endothelial origin and purity was verified by immunocytochemistry using anti-mouse CD31 antibody (Dako, Heverlee, Belgium).

### Assessing the cytotoxic effects of perfringolysin on bovine endothelial cells

In order to visually assess cytotoxicity, BUVEC were seeded on 13-mm-circular glass slides (VWR International BVBA, Leuven, Belgium) in a 24-well plate at a concentration of 1 × 10^5^ cells/mL and were incubated at 37 °C in the presence of 5% CO_2_. After 36 h, the cells were exposed to 3% filter-sterilized supernatant of an overnight culture diluted in serum-free endothelial cell growth medium (SFM). The strains used for producing the supernatants were the wild-type strain JIR325 and its isogenic mutants (Table 1). SFM was used as a control. After 1.5 h incubation, the cells were rinsed three times with HBSS containing Ca^2+^ and Mg^2+^. They were fixed with 100% methanol and stained with Haemacolor-stain (Merck, Darmstadt, Germany). The glass slides were mounted on a microscope slide and observed microscopically.

To quantify the cytotoxicity, BUVEC were seeded in a 96-well plate at a concentration of 1 × 10^5^ cells/mL and incubated for 36 h as described above. 1% or 6% of the above mentioned supernatants in SFM was added to the cells. SFM was used to determine reference values. After 1.5 h incubation, a Neutral Red Uptake Assay was performed [29]. Briefly, Neutral Red Medium (EGM-2 medium containing neutral red (Merck N.V./S.A., Overijse, Belgium)) was added to the cells and the plates were incubated at 37 °C. After 3 h of incubation, the cells were rinsed with HBSS containing Ca^2+^ and Mg^2+^ and treated with extracting solution (50% absolute ethanol, 49% distilled water and 1% glacial acetic acid) for 15 min at room temperature on a shaker to extract the neutral red from the cells. The absorbance was determined at 550 nm and cell viability was expressed as the percentage of viable cells compared to untreated cells (negative control) and cells treated with the supernatant of the wild-type (positive control). Each culture supernatant was tested in duplicate in three independent assays.

### Statistical analysis

Significant differences in the number of loops with necrosis between the wild-type and the mutants were determined using a two-tailed Fisher Exact test (*P* < 0.05). Significant differences between the relative viability of the mutants and 0%, which corresponds with the relative viability of the wild-type strain, were investigated using a two-tailed Wilcoxon signed rank test (*P* < 0.05). A one-way analysis of variance (ANOVA) with the post hoc Tukey-Kramer multiple-comparison test was used to identify significant differences in the relative viability of cells after contact with the supernatants of the mutants (*P* < 0.001). All statistical analyses were performed using GraphPad Prism Software 5.0 (GraphPad Software, Inc., USA).

## Results

### Assessing the role of perfringolysin by using an intestinal loop model

**Figure 1:**
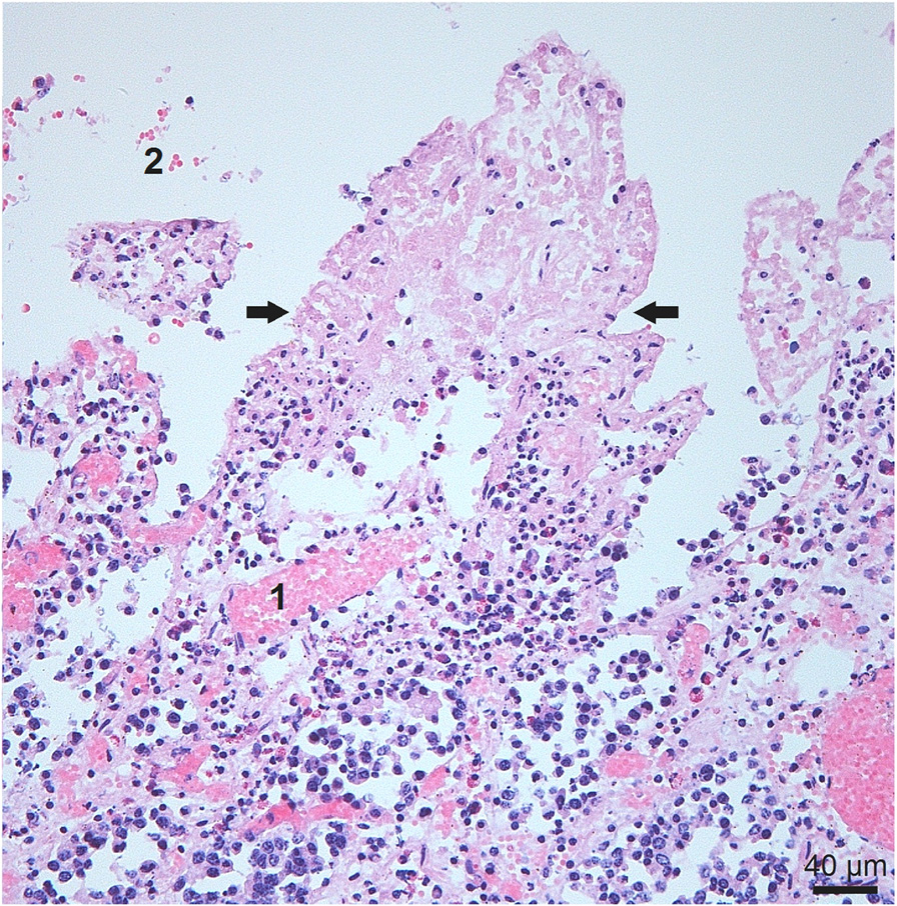
**Histological section from an intestinal loop inoculated with wild-type.** Hematoxylin and eosin stained histological section from an intestinal loop inoculated with a logarithmic culture of the wild-type strain and milk replacer, sampled after 6 h incubation. Arrows indicate the demarcation of the necrosis of the villus tip with loss of epithelial cells. Also capillary congestion (1) and hemorrhage (2) are present.

**Figure 2:**
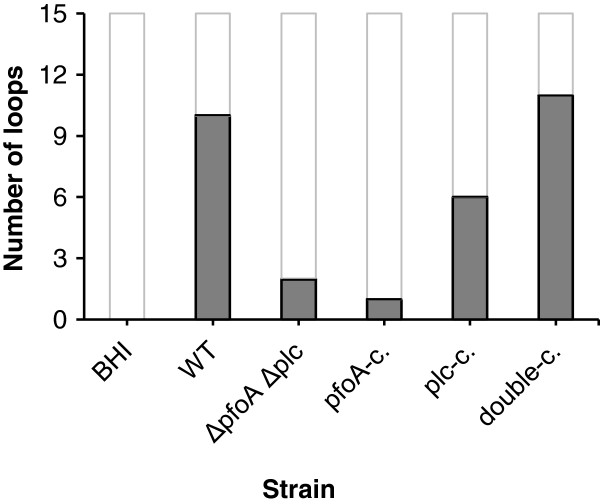
**Number of loops with necrosis for wild-type and isogenic mutants.** The graph shows the total number of loops in which necrosis was present, evaluated 6 h after inoculation with a logarithmic-phase culture of the wild-type strain (WT), Δ*pfoA* Δ*plc* mutant (Δ*pfoA* Δ*plc*), *pfoA*-complemented Δ*pfoA* Δ*plc* mutant (*pfoA*-c.), *plc*-complemented Δ*pfoA* Δ*plc* mutant (*plc*-c.), double-complemented Δ*pfoA* Δ*plc* mutant (double-c.) and BHI as a control in three calf intestinal loop assays. Each strain was inoculated in quintuplicate. A significant difference was found between the wild-type and the Δ*pfoA* Δ*plc* double mutant (*P* < 0.01) and the *pfoA*-complemented Δ*pfoA* Δ*plc* mutant (*P* < 0.01).

Histological examination of loops inoculated with the wild-type strain showed necrosis of the tips of the villi and congestion of the capillaries and hemorrhages in the underlying viable tissue (Figure 1). Rod-shaped bacteria were found attached to cellular debris in the lumen and to the mucosa. To elucidate the relative contribution of perfringolysin and alpha toxin in the induction of necrohemorrhagic enteritis a wild-type strain and its Δ*pfoA* Δ*plc* mutant was used in combination with the Δ*pfoA* Δ*plc* mutant complemented with either the *pfoA* or the *plc* gene or both (Figure 2). Using the same intestinal loop model, Valgaeren et al. observed mucosal hemorrhages, not only in the challenge strain inoculated loops, but also in some control loops. Necrosis only occured after inoculation of a *C*. *perfringens* strain [20]. Therefore, in the present study we focussed on the presence of necrosis to compare the mutants with the wild-type strain. Significantly fewer loops injected with the Δ*pfoA* Δ*plc* mutant or the *pfoA*-complemented Δ*pfoA* Δ*plc* mutant (or *plc*-deficient strain) showed necrosis in comparison to the wild-type strain (*P* < 0.01). The *plc*-complemented Δ*pfoA* Δ*plc* mutant (or *pfoA*-deficient strain) could induce necrotic lesions in fewer loops as compared to the wild-type strain, but the difference in number of necrotic loops did not reach statistical significance. The double-complemented Δ*pfoA* Δ*plc* mutant showed necrosis in as many loops as the wild-type. Apparently, only the complementation of both toxins could restore the activity to that of the wild-type. In the control loops no necrotic lesions were detected.

### Assessing the cytotoxic effects of perfringolysin on bovine endothelial cells

**Figure 3:**
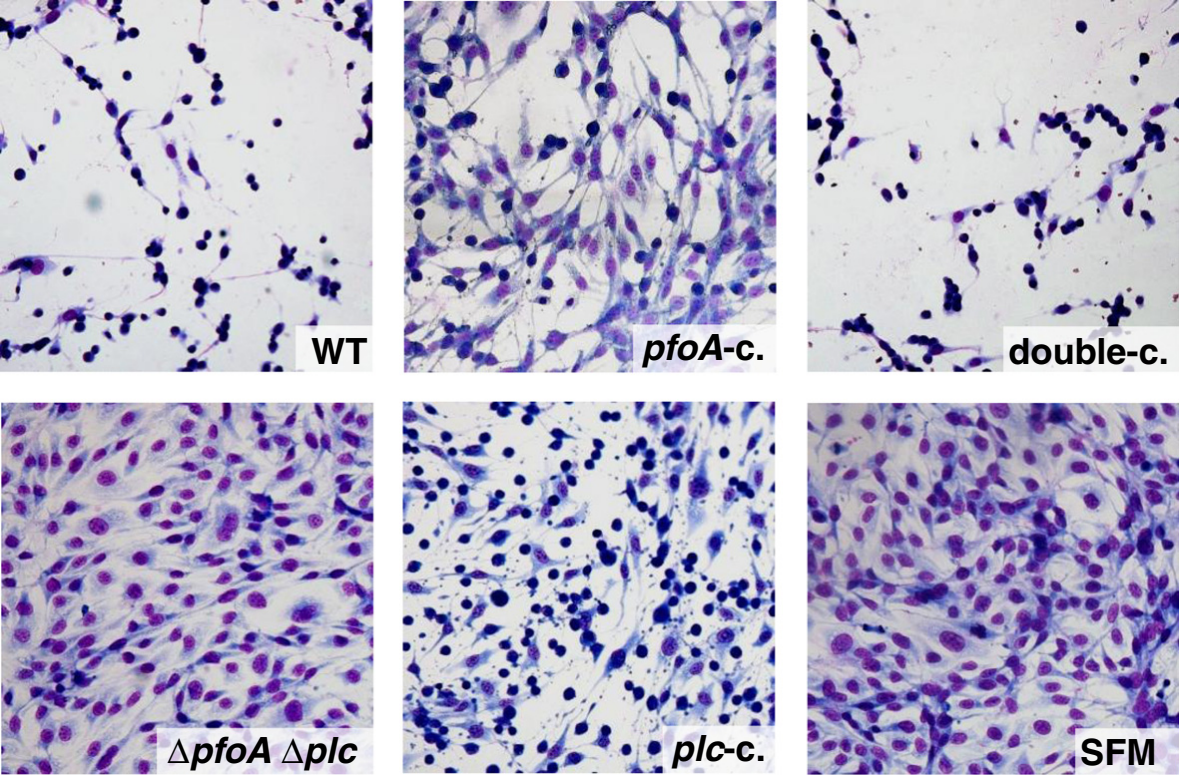
**Cytotoxic effect of the supernatant of wild**-**type and isogenic mutants on bovine umbilical vein endothelial cells** (**BUVEC.**) Photomicrograph of Haemacolor-stained cells after 1.5 h exposure to 3% supernatant of the wild-type strain (WT), Δ*pfoA* Δ*plc* double mutant (Δ*pfoA* Δ*plc*), *pfoA*-complemented Δ*pfoA* Δ*plc* mutant (*pfoA*-*c*.), *plc*-complemented Δ*pfoA* Δ*plc* mutant (*plc*-c.), double-complemented Δ*pfoA* Δ*plc* mutant (double-c.) and SFM as a negative control. Magnification, ×400.

To determine whether perfringolysin and alpha toxin have cytotoxic effects on endothelial cells, bovine umbilical cord endothelial cells were exposed to supernatant of a wild-type strain and its isogenic mutants. Almost all cells detached after exposure to supernatant of the wild-type strain (Figure 3). No cytotoxicity could be observed visually when using the supernatant of the Δ*pfoA* Δ*plc* mutant as compared to the control. The supernatants of the *pfoA*-complemented Δ*pfo* Δ*plc* mutant and the *plc*-complemented Δ*plc* Δ*pfo* mutant were less cytotoxic in comparison with supernatant of the wild-type strain, but more cytotoxic as compared to supernatant of the Δ*plc* Δ*pfo* mutant. Complementation of the Δ*plc* Δ*pfo* mutant with both the *plc* and *pfo* gene restored cytotoxic effects to the wild-type levels. These data indicate that perfringolysin and alpha toxin are important factors for cytotoxicity on bovine endothelial cells and have a synergistic effect.

**Figure 4:**
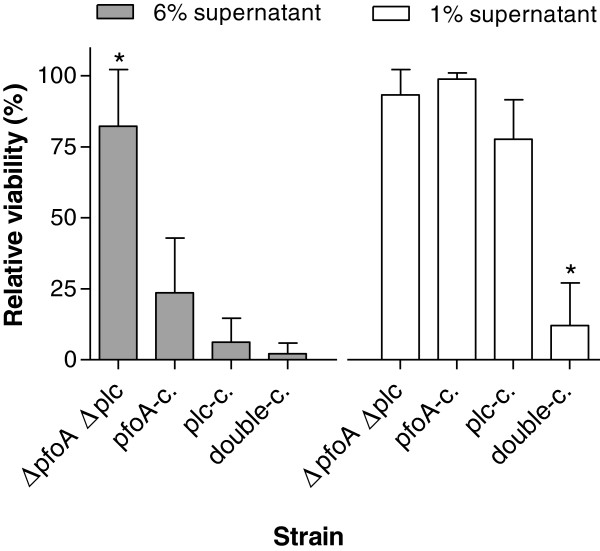
**Effect of the supernatant of wild**-**type and isogenic mutants on the viability of bovine umbilical vein endothelial cells.** The graph shows the percentage of viable cells relative to viable untreated cells (negative control) and cells treated with supernatant of the wild-type strain (positive control) after 1.5 h incubation with 6 and 1% supernatant of Δ*pfoA* Δ*plc* double mutant (Δ*pfoA* Δ*plc*), *pfoA*-complemented Δ*pfoA* Δ*plc* mutant (*pfoA*-c.), *plc*-complemented Δ*pfoA* Δ*plc* mutant (*plc*-c.) and double-complemented Δ*pfoA* Δ*plc* mutant (double-c.). The values are the average of three independent experiments conducted in duplicate with error bars representing the standard deviations (SD). Asterisks indicate significant difference between that strain and all other strains for each concentration (*p* < 0.001).

To quantify the relative contribution of alpha toxin and perfringolysin on endothelial cell cytotoxicity, the viability was assessed by a Neutral Red Uptake Assay. The percentage viability after 1.5 h incubation with 1 and 6% supernatant of the isogenic mutants relative to viable untreated cells (negative control) and cells treated with supernatant of the wild-type strain (positive control) are shown in Figure 4. The cytotoxic effect of 6% supernatant of the Δ*pfoA* Δ*plc* mutant was significantly lower than the wild-type (*P* < 0.05) and all other tested strains (*P* < 0.001). The cytotoxicity of 1% supernatant of Δ*pfoA* Δ*plc* mutant, *pfoA*-complemented Δ*pfoA* Δ*plc* mutant and the *plc*-complemented Δ*pfoA* Δ*plc* mutant was significantly lower as compared to that of the wild-type (*P* < 0.05) and that of the double-complemented Δ*pfoA* Δ*plc* mutant (*P* < 0.001). These results confirm that perfringolysin as well as alpha toxin contribute to the cytotoxic effect of a *C*. *perfringens* culture, and show a synergistic effect of perfringolysin and alpha toxin.

## Discussion

The presence of a causative toxin in a *C*. *perfringens* strain determines its potential to cause lesions and subsequently diseases in several animal species. However, there is still controversy on the toxin responsible for bovine necrohemorrhagic enteritis. In a previous study, it was demonstrated that type A strains from bovine and non-bovine origin can induce necrohemorrhagic lesions in a calf intestinal loop assay [20]. These results suggest that the causative toxin is one of the toxins produced by all *C*. *perfringens* type A strains. In the present study, it was shown that perfringolysin and alpha toxin are involved in the induction of necrohemorrhagic lesions. Indeed, a *plc*-complemented Δ*pfoA* Δ*plc* mutant (or *pfoA*-deficient strain) and a *pfoA*-complemented Δ*pfoA* Δ*plc* mutant (or *plc*-deficient strain) of a gas gangrene strain had a decreased ability to induce necrohemorrhagic lesions in a calf intestinal loop assay. The gas gangrene strain JIR325 was used, because perfringolysin- and alpha toxin-deficient mutants of this strain were already available and because this strain was able to induce mucosal hemorrhages and necrosis of the villus tips comparable to the lesions induced by bovine strains in a calf intestinal loop model [20]. In previous studies, it was suggested that epsilon toxin and beta2-toxin are essential in the development of necrosis. Filho *et al*. induced lesions in a calf intraduodenally inoculated with a type D strain [15]. The authors proposed epsilon toxin as causative toxin. However, based on our results, alpha toxin and perfringolysin may also have been involved in lesion development, since both toxins are produced by type D strains as well. In an intestinal loop assay, comparing alpha and epsilon toxin, only epsilon toxin was able to cause severe oedema and hemorrhages in the lamina propria [9], but these authors did not observe necrosis, in contrast to our results which showed necrohemorrhagic lesions comparable to field cases. Manteca et al. stated that beta2-toxin was an essential toxin, because inoculation of a beta2-positive type A strain into a bovine ligated intestinal loop caused necrohemorrhages of the intestinal wall [19]. However, the strain also produced a high level of alpha toxin and the authors proposed a synergistic effect of alpha toxin and beta2-toxin. Since nearly all type A strains produce perfringolysin as well, this toxin may also have been important in the development of the lesions. In addition, our results agree with a recent study in which only beta2-negative type A strains were isolated from calves with necrotic enteritis and these strains were able to induce pathologic changes when inoculated in intestinal loops [10]. It is still possible that beta2-toxin or other toxins can have supplementary effects.

Valgaeren et al. also found indications that endothelial damage may be involved in the early stages of intestinal lesion development [20]. Our results suggest perfringolysin-induced cytotoxic effects on endothelial cells may play a potential role in the development of necrohemorrhagic enteritis. We showed that the *plc*-complemented Δ*pfoA* Δ*plc* mutant (or *pfoA*-deficient strain) was significantly less cytotoxic for bovine umbilical cord endothelial cells (BUVEC). Endothelial cells form a vital barrier that controls the exchange of cells, macromolecules and fluids between the vascular lumen and the surrounding tissue. They also maintain the normal blood flow due to their antiplatelet, anticoagulant and fibrinolytic properties. Disruption of the endothelial barrier leads to increased vascular permeability along with tissue edema and hemorrhage. Furthermore, it augments local coagulation and vascular thrombosis, and subsequent hypoxic tissue necrosis [30]. In order to confirm that the endothelium is the target cell of perfringolysin or alpha toxin in bovine necrohemorrhagic enteritis, it would be interesting to localize the toxin in lesions, as already been done for beta toxin in necrotic enteritis in piglets and in a human case [31,32]. Beta toxin has been shown to induce porcine endothelial cell damage in vitro and to bind to endothelial cells, and not to epithelial cells, in the gut of diseased animals, suggesting that disruption of endothelial cells plays a role in type C enteritis [30,31,33].

Additionally, our results show that perfringolysin and alpha toxin act synergistically in inducing BUVEC cytotoxicity and necrohemorrhagic lesions in a calf intestinal loop model. In gas gangrene, perfringolysin and alpha toxin also act synergistically [22-24,34]. Alpha toxin is a phospholipase C that hydrolyzes phosphatidylcholine and sphingomyelin, both of which are important constituents of eukaryotic cell membranes. Perfringolysin is a cholesterol-dependent cytolysin and oligomerizes upon contact with cholesterol-containing membranes to form large transmembrane pores by inserting a beta-barrel into the membrane [24]. It has been stated that the ability of perfringolysin to perforate the membrane of target cells, is determined by the amount of free cholesterol molecules present [35,36]. Moe and Heuck found that alpha toxin cleaves the phosphocholine headgroup of phosphatidylcholine, increasing the number of free cholesterol molecules in the membrane and by doing so, facilitating the interaction of perfringolysin and cholesterol [36]. This concerted action of alpha toxin and perfringolysin may contribute to the synergistic effect between both toxins in gas gangrene and in bovine necrohemorrhagic enteritis.

In gas gangrene, perfringolysin modulates the host inflammatory response by upregulating leukocyte and endothelial adhesion molecules. This causes leukocyte accumulation within the blood vessels and inhibits the normal influx of phagocytic cells into infected host tissue, reducing inflammation [22,23,37]. Additionally, alpha toxin enhances the expression of platelet adhesion molecules, contributing to the formation of freely moving intravascular aggregates of platelets, fibrin and neutrophils. This leads to the obstruction of the vessels and contributes to a decreased blood flow [34]. Gas gangrene is characterized by tissue necrosis, thrombosis and a lack of leukocyte infiltration at the site of infection. On the contrary, bovine necrohemorrhagic enteritis is associated with congestion of the capillaries, hemorrhages and inflammation [7,10,20]. So perfringolysin and alpha toxin appear to be involved in both diseases, but may act in a different way. The use of mutants deficient in the production of alpha toxin or perfringolysin in a mouse myonecrosis model showed that alpha toxin is essential for thrombosis formation [22,23]. Furthermore, when rabbits were treated intravenously with recombinant perfringolysin a vasodilatory effect and a reduced systemic vascular resistance was observed. On the other hand, in rabbits treated with recombinant alpha toxin the vascular resistance was maintained and the arterial pressure was reduced [23,38,39]. Altered vascular integrity, but not vascular occlusion, seems to be in accordance with the role of perfringolysin in bovine necrohemorrhagic enteritis. This may also explain partly the inflammation present in necrohemorrhagic enteritis as opposed to the lack of leukocyte infiltration in gas gangrene.

Next to the toxic effect of perfringolysin on endothelial cells, other effects on the gastro-intestinal mucosa are most likely of importance in the development of necrohemorrhagic enteritis. Indeed, before perfringolysin can target the endothelial cells, it has to cross the epithelial barrier. While cytotoxic effects of perfringolysin and alpha toxin on intestinal epithelial cells cannot be excluded, also other *C*. *perfringens* toxins, enzymes or other molecules could affect the intestinal integrity. Intestinal integrity disturbances can also be caused by *C*. *perfringens* independent factors in the field, such as viral and parasitological pathogens. The most well-known example of a predisposing pathogen for necrotic enteritis is coccidiosis in broilers [40,41], but also in calves several infectious agents, such as coccidia, enteropathogenic bacteria, corona- and rotaviruses can affect the intestinal barrier integrity [40-44]. In addition, certain feed components can act as predisposing factors for the induction of gut lesions. These include high non-starch polysaccharide containing diets and high protein diets, the latter most likely feeding the auxotrophy *C*. *perfringens* has for many amino acids [45,46]. The diet can have a direct effect on the virulence of *C*. *perfringens*, but it can as well affect the intestinal tract. In calves fed with milk replacing proteins an increase in permeability of the intestinal mucosa was observed, which caused leakage of macromolecules from the gut into the tissues [41,47-49]. This might also facilitate the uptake of toxins through the epithelial barrier.

In conclusion, our study indicates that perfringolysin is involved in the pathogenesis of bovine necrohemorrhagic enteritis and acts synergistically with alpha toxin. We hypothesize that both toxins may induce intestinal lesions by targeting the endothelial cells.

## Competing interests

The authors declare that they have no competing interests.

## Authors’ contributions

SV, EG, BV, BP, LT, RD, PD and FVI participated in the design of the study. SV, EG, BV, BP, KV, SS and RD performed the calf intestinal loop assays. SV carried out the cell cytotoxicity assays and analyzed the data. SV, FH, RD, PD and FVI wrote the manuscript. All authors revised the manuscript and read and approved the final manuscript.
